# Supplementary material for: Qualitative Analysis of a Twitter-Disseminated Survey Reveals New Patient Perspectives on the Impact of Urinary Tract Infection
Source: Antibiotics (Basel). 2022 Nov 23;11(12):1687. doi: 10.3390/antibiotics11121687 (PMC9774672; doi:10.3390/antibiotics11121687)
Supplement: Supplementary file 1 [file antibiotics-11-01687-s001.zip › antibiotics-1971665-supplementary.pdf]

UTIGA Codebook

Code Name	Description	References
Q1. Dependency		1
Antibiotics		31
Bathroom		66
Medical system		9
Dependency Sum		107
Q1. Diagnosis		
Chronic UTI		85
Interstitial cystitis		12
Recurrence		43
Q1. Economic Impact		5
Healthcare costs		24
Income loss	Only if specifically mentioned, not implied from listing 'job loss'	7
Economic Sum		36
Q1. Limitations-Impact		16
Diet	Eating/drinking habits	38
Exercise		40
Overall functioning	Includes impact on general activities/activities of daily living	113
Sleep		40
Travel	Travel and ability to be away from the house	21
Doctors appts		6

Code Name	Description	References
Leave the house		51
Travel for pleasure		8
Unpredictability		56
Work/School	Ability to work and job performance	82
Career-promotion loss		30
Sum Work/school		112
Q1. Mental health		66
Anxiety	Includes ‘worry’	72
Depression		50
Suicidal Ideation		29
Distress	more extreme than stress, with desperation component	15
Embarrassment		11
Fear		41
Frustration		45
Lowered self-esteem		8
Resignation		3
Stigma	Related to chronic-pain	3
Stress		17
Q1. Quality of life		9
Consuming		41
Moderate		27
Severe		51

Code Name	Description	References
Sum of Quality of Life		128
Q1. Physical health		28
Fatigue		27
Malaise	Unwell	15
Weight gain		5
Pain	If not directly in reference to UTI symptoms	123
Location		10
Severity		54
Type		0
Acute		6
Chronic		89
Sum pain		282
Q1. UTI Complications		7
Ability to have children		13
Hospitalization		11
Miscarriage	Due to infection	3
Sepsis		3
Q1. Sexual health		69
Q1. Social health		27
Enjoyment	Includes general reference to ‘holiday’ but excludes ‘travel for holiday’	28
Family or home life		51
Spouse/significant other	Impact on relationship with spouse	31

Code Name	Description	References
Personal relationships		54
Sum of social health		191
Q1. UTI Symptoms		43
Bladder pain-pressure		26
Dysuria		19
Frequency		41
Incontinence		6
Other		18
Rigors or chills		1
Urgency		20
Q1. Treatment experience		52
Antibiotics		68
Antibiotic side effects		26
Duration		92
Long-term		28
Prescription hesitancy	This captures unwillingness of doctors/fear of prescribing longer term antibiotics.	4
Antibiotic Resistance		28
Limited medications for treatment		13
Medical system	Negative experience	19
Recurrence	Recurrence or treatment failure	16
Recurrence-chronic UTI	Recurrence or treatment failure, chronic UTI, embedded UTI	48
TVT Mesh		12

Code Name	Description	References
Uncertainty of cure		29
Unclear results		25
Q2. Clinician experience		28
Inadequate management or care	Examples in terms of diagnosis and/or treatment, not ordering appropriate diagnostics, not treating appropriately, more thorough diagnostics/workup, lack of follow-up	129
Interaction	Includes dismissive, lack of listening, not believing	153
Empathy		9
Sum Interaction/Empathy		162
Named provider	If respondent specifically names a certain doctor	26
No belief in embedded		16
Not treating symptoms		21
Stewardship-AMR-fears		11
Q2. Accessibility	For tests, appointments, clinicians, travel distances, timeliness of access	
Diagnostics or treatment		42
Chronic UTI Specialist		43
Timeliness		15
Travel		12
Q2. Awareness-Knowledge-Beliefs	In general	22
Doctors-medical system	Lack of knowledge and or awareness; general UTI	109
Acknowledge UTI or condition as important	Take seriously, value, respect	55
Chronic UTI	general	2
Disbelief in chronic UTI		13

Code Name	Description	References
Lack of chronic UTI knowledge		33
Incorporating current research or treatment	Accepting, entertaining, acknowledging new research	11
Initiating own research; digging deeper into patient diagnosis		10
Knowledge related to testing inaccuracies		28
Sum UTI knowledge doctors/medical sys.		184
Scientists		4
Personal		17
Public		16
Q2/Q3. Women's health issue	Neglected area due to women's health issue	52
Q2/Q3.Combined Guidelines	Shortcomings of UTI guidelines	52
Q3. Diagnostics		115
PCR testing		4
Standard urine culture		24
Sum Diagnostics	Shortcomings	143
Misdiagnosis with IC or other cond.		21
Q3. Research		53
Impact on quality of life	Research on QoL but also general statements about QoL impacts	38
Menopause	With respect to UTI	5
Acceptance of chronic UTI as dx	Acceptance/recognition as a diagnosis and treatment and or more research to 'prove' chronic UTI exists	17
General Research on Chronic UTI		38
Mechanistic causes of Chronic UTI	Genetic, microbiome, causative organisms, acute vs chronic	15

Code Name	Description	References
Sum of Mechanistic + general chronic UTI		52
Microbiome-Urobiome		18
Pathophysiology	General pathophysiology category	28
Questions about UTI	Or biofilms, recurrent UTI Or statements related to lack of information	10
Vaginal Mesh		9
Q3. Support-Encouragement-Imploring help		125
Q3. Treatment		107
Chronic UTI Tx		38
Cure		48
Duration		4
Natural remedies		5
Pain treatment		12
Prevention		20
Targeted bladder wall treatment		9
Vaccine		4
